# Catheter Ablation in Persistent AF, the Evolution towards a More Pragmatic Strategy

**DOI:** 10.3390/jcm10184060

**Published:** 2021-09-08

**Authors:** Louisa O’Neill, Jean-Yves Wielandts, Kris Gillis, Gabriela Hilfiker, Jean-Benoit Le Polain De Waroux, Rene Tavernier, Mattias Duytschaever, Sebastien Knecht

**Affiliations:** Department of Cardiology, AZ Sint-Jan Hospital, 8200 Bruges, Belgium; Jean-Yves.Wielandts@azsintjan.be (J.-Y.W.); Kris.Gillis@azsintjan.be (K.G.); Gabriela.Hilfiker@azsintjan.be (G.H.); Jean-Benoit.LePolainDeWaroux@azsintjan.be (J.-B.L.P.D.W.); rene.tavernier@azsintjan.be (R.T.); Mattias.duytschaever@azsintjan.be (M.D.); sebastien.knecht@azsintjan.be (S.K.)

**Keywords:** persistent AF, pulmonary vein isolation, substrate ablation, AF mapping, novel ablation technology

## Abstract

Atrial fibrillation (AF) is the most common cardiac arrhythmia worldwide and represents a heterogeneous disorder with a complex pathological basis. While significant technological advances have taken place over the last decade in the field of catheter ablation of AF, response to ablation varies and long-term success rates in those with persistent AF remain modest. Mechanistic studies have highlighted potentially different sustaining factors for AF in the persistent AF population with substrate-driven focal and re-entrant sources in the body of the atria identified on invasive and non-invasive mapping studies. Translation to clinical practice, however, remains challenging and the application of such mapping techniques to clinical ablation has yet to demonstrate a significant benefit beyond pulmonary vein isolation (PVI) alone in the persistent AF cohort. Recent advances in catheter and ablation technology have centered on improving the durability of ablation lesions at index procedure and although encouraging results have been demonstrated with early studies, large-scale trials are awaited. Further meaningful improvement in clinical outcomes in the persistent AF population requires ongoing advancement in the understanding of AF mechanisms, coupled with continuing progress in catheter technology capable of delivering durable transmural lesions.

## 1. Introduction

Atrial fibrillation (AF) represents the most common cardiac arrhythmia worldwide and is associated with a significant burden of morbidity and mortality. The incidence of AF has increased dramatically over the last two decades and current prevalence is expected to further double between 2010 and 2060 with a predicted 17.9 million adults living with AF by this timepoint [1]. AF is traditionally classified according to the characteristic duration of episodes experienced. Paroxysmal AF describes episodes terminating spontaneously or with intervention within seven days. AF that is sustained longer than seven days is considered persistent, while long-standing, persistent AF refers to continuous AF lasting >1 year [2]. It is generally accepted, however, that this classification scheme does not accurately reflect the diversity of the underlying pro-arrhythmic atrial substrate in patients with AF. The role of catheter ablation as a rhythm control strategy in AF is well established. In contrast to paroxysmal AF patients, in whom one-year success rates are reported as high as 90% with current workflows [3], outcomes are significantly more modest in persistent AF, with many patients requiring multiple procedures to maintain sinus rhythm. In these patients, progressive structural and electrical remodeling creates a substrate for the initiation and maintenance of AF in the body of the left atrium. As such, more extensive ablation strategies have been advocated in the persistent AF cohort including linear ablation, ablation of complex fractionated electrograms, and rotor and voltage-based ablation [4,5,6,7]. In this review we provide an overview of the major ablation strategies utilized in the last two decades, we discuss developments in AF mapping and its application to clinical ablation and we highlight contemporary advances in ablation technology demonstrating promise for the creation of durable lesion sets in the persistent AF population.

## 2. Catheter Ablation—The Historical Perspective

The observation by Haissaguerre et al. nearly two decades ago that AF may be initiated by focal pulmonary vein triggers led to the development of the pulmonary vein isolation (PVI) radiofrequency (RF) ablation procedure, the endpoint of which is electrical disconnection of the pulmonary veins [8]. PVI represents the cornerstone of AF ablation worldwide with current ESC guidelines recommending it as a primary strategy for all AF patients undergoing ablation (class I, level of evidence A) [2]. Indeed, a 2015 survey reported that PVI alone is performed for first-time persistent AF ablation in 67% of 30 European centres with one-year success rates off anti-arrhythmic drugs in the range of 50–60% [9]. It is important to view the current emphasis on PVI alone in the context of the historical evolution of catheter ablation for persistent AF as it represents a return towards a simplified workflow following the evaluation of various adjunctive strategies over the last two decades.

Although the majority of studies evaluating the technique of PVI, evolving from targeted ablation of ectopy within the pulmonary veins to wide area circumferential ablation, focused on paroxysmal AF patients, several assessed this approach in persistent AF patients. An early study of segmental isolation of the pulmonary veins including a small number of persistent AF patients demonstrated significantly lower success rates at one year compared to those with paroxysmal AF, with over 50% experiencing recurrence [10]. Although better outcomes were seen in subsequent larger studies, results were largely underwhelming, with freedom from AF in the range of 58–69% at ≥1 year, and higher recurrence rates in persistent vs. paroxysmal AF patients being a frequent finding [11,12,13].

The inferior results seen with PVI alone in the persistent AF cohort are likely to reflect substrate-driven AF, in the setting of more advanced atrial remodeling, as distinct from trigger-driven AF in early paroxysmal AF which responds well to PVI. Conduction velocity heterogeneity and altered tissue refractoriness consequent on progressive atrial fibrosis and dilatation increase vulnerability to the development and maintenance of AF [14,15,16,17]. Furthermore, left atrial fibrosis burden (measured on cardiac MRI imaging and invasive voltage mapping) and dimensions are associated with persistence of AF [18,19,20,21] and recurrence post ablation [22,23,24,25]. As such, ablation techniques targeting this arrhythmia substrate were proposed as adjunctive strategies to PVI in persistent AF patients.

### 2.1. The Advent of Linear Ablation

The motivation for left atrial linear ablation was based on experience with the surgical maze procedure involving atrial compartmentalization through the creation of lines of electrical block, theoretically reducing the ability of the atria to sustain re-entry circuits responsible for AF maintenance [26].

The Bordeaux group first described the technique of linear ablation at the mitral isthmus and the left atrial roof as an adjunct to PVI in 2004 [4,27], the endpoint of which was bidirectional block across the ablated lines. Subsequent prospective and randomized studies of linear ablation, as an adjunct strategy to PVI, reported significantly higher rates of sinus rhythm on follow-up compared to PVI alone, an effect that was more pronounced in patients with persistent compared to paroxysmal AF [28,29,30]. Achievement of block at the mitral isthmus was variable in these studies; however, with rates of complete mitral isthmus block as low as 31%, although the rate of roofline block was demonstrably higher. Furthermore, while evidence suggests that recurrence of atrial tachycardia tends to occur less frequently in those undergoing adjunct linear ablation vs. PVI alone, incomplete block across ablation lines has been identified as a significant risk factor for the development of atrial tachycardia on follow up [31]. Overall, while several studies appear to support an incremental benefit to linear ablation in persistent AF patients, results are generally conflicting with disappointing long-term outcomes seen in others [29,32].

### 2.2. Box Isolation as a Form of Linear, Endpoint Driven Ablation

A linear ablation strategy of ‘box isolation’ of the posterior left atrial wall, consisting of a roof and an inferior transverse line joining the encircled pulmonary veins was described by Kumagai et al. in 2007 [33] with the endpoint of complete electrical isolation of the region bound by the ablation set. The rationale behind this approach was the isolation of focal and re-entrant AF drivers frequently described at the left atrial posterior wall [34], reflecting its common embryologic origin with the pulmonary veins, and the reduction in the conducting size of the atria. Supporting this point, a mechanistic study of phase mapping in patients with persistent AF demonstrated both a reduction in atrial critical mass and the number of AF drivers in the body of the atrium post box isolation [35]. Conflicting reports exist regarding the efficacy of this approach, however, and several small randomized studies have failed to demonstrate a benefit to the box isolation strategy [36,37,38].

### 2.3. CFAE Ablation

Distinct from endpoint driven PVI and linear ablation, the ablation of complex fractionated atrial electrograms (CFAE) represents a strategy that gained significant traction over the last two decades. CFAEs can be defined as electrograms displaying continuous electrical activity, short mean cycle lengths or complex fractionated potentials [39]. The justification for targeting these for ablation is based on their association with sites of slow conduction or block, anchor points for re-entry circuits, wave break, and wavefront collision and ganglionated plexi [5,40,41]. Mapping and identification of CFAEs in sinus rhythm or AF are facilitated by automated algorithms incorporated into contemporary electro-anatomic mapping systems. CFAE ablation was initially proposed by Nademee et al. in 2004 as a stand-alone ablation strategy with excellent outcomes reported in paroxysmal and persistent AF patients undergoing ablation of sites identified during mapping in AF [5]. A subsequent study in 2007, however, failed to reproduce these results in persistent AF patients with only 33% remaining in sinus rhythm at >1 year follow up [42]. As such CFAE ablation was evaluated as an adjunct to PVI in the persistent AF population with mixed results. Oral et al. failed to show an outcome benefit of incremental CFAE ablation after PVI in long-standing persistent AF [43], findings echoed by the RADAR-AF multicentre randomized trial in 2014 [44]. A randomized study by Elayi et al., including 144 patients with permanent AF, demonstrated superior, albeit modest results with additional CFAE ablation with 68% freedom from atrial arrhythmia at 16 months [45]. Furthermore, a meta-analysis of seven randomized and non-randomized trials reported a significantly higher rate of sinus rhythm at 12 months when CFAE ablation was performed in addition to PVI in persistent but not paroxysmal AF patients [46] highlighting the differences in AF mechanisms and substrate between these two populations. Of note, however, several of the studies included in this meta-analysis evaluated a combination strategy of linear and CFAE ablation limiting the ability to assess the added value of CFAE ablation alone. Additional factors to consider when reviewing these studies include variability in the definition of CFAEs and endpoints which renders it difficult to compare results between them. Furthermore, the role of CFAEs in the maintenance of AF remains unclear with a lack of correlation previously demonstrated between sites exhibiting CFAEs during AF compared to sinus rhythm [47] as well as a poor association between CFAE sites and local complex activation or re-entry activity in AF [48]. These and other mechanistic studies suggest that many CFAEs may be passive, rather than active, participants in AF perpetuation [49] potentially leading to extensive ablation of noncritical areas, without a clear endpoint, as well as prolonged procedure times.

### 2.4. The Stepwise Approach to Ablation

A ‘stepwise’ approach for ablation combining CFAE and linear ablation in persistent AF patients was first described by Haissaguerre et al. in 2005 [50,51]. This involved isolation of the pulmonary veins, ablation of complex fractionated atrial activity and linear ablation at the left atrial roof and mitral isthmus, with AF termination as a procedural endpoint. In initial reports, this strategy resulted in termination of AF in 87% with notably high rates of sinus rhythm (95%) at 11 months after 1 or more procedures in persistent AF patients [50]. In 2011, Rostock et al. evaluated this approach in 395 persistent AF patients and reported a success rate of 79% after a median of 2.3 procedures and 27 months of follow-up [52]. Subsequently, in 2015, Scherr et al. reported arrhythmia-free survival rates after a mean of 2.1 procedures of 89.7%, 79.8%, and 62.9%, at 1, 2, and 5 years respectively in 150 persistent AF patients undergoing a stepwise approach [53]. Those advocating this strategy in the persistent AF cohort would argue that high success rates can be achieved with extensive substrate ablation to sinus rhythm over several procedures, although efficacy tends to diminish with time on follow-up.

The multicentre STAR AF II trial published in 2015 represents the most robust evaluation of ablation strategy in persistent AF patients to date [32]. In this trial, 589 patients were randomized to PVI vs. PVI plus CFAE ablation or PVI plus linear ablation at the left atrial roof and mitral isthmus. At 18 months no difference was seen in outcomes between the three ablation strategies with 59% in the PVI arm free of recurrent atrial fibrillation, compared to 49% and 46% in the CFAE and linear ablation arms respectively (*p* = 0.015) again underscoring the modest results obtainable in this patient group. This study did not evaluate the stepwise approach, however, and in those receiving linear ablation as an adjunct strategy block across both lines was achieved in only 74% of patients.

Despite the abundance of studies evaluating different ablation techniques in the persistent AF population, there remains a lack of consensus regarding the optimum strategy in this cohort and a strong suggestion from STAR AF II that ablation beyond PVI may not be of benefit. As such, the central role of PVI is underscored in the 2020 ESC guidelines while the employment of additional lesions beyond PVI may be considered but are ‘not well established’ and carry a class IIb recommendation [2].

## 3. Mapping of AF

### 3.1. The Principle of Phase Mapping

In parallel with the evaluation of different ablation techniques, a drive towards a better understanding of AF mechanisms resulted in the emergence of two major invasive and non-invasive forms of AF mapping technology, namely ‘Focal impulse and rotor modulation’ or FIRM mapping and electrocardiographic imaging or ECGi mapping. These techniques employ the principle of phase mapping, a mathematical approach for the assessment of spatial and temporal periodicity in tissue and identification of periodic rotations or ‘rotors’ [54]. In mechanistic terms, a rotor is described as a form of functional re-entry where the wavefront and wavefront tail meet at an area known as the phase singularity [55]. Optical mapping work in animal models provides compelling evidence for the existence of rotors and their role in AF perpetuation [56] and it is from insights derived from such studies that the clinical translation to AF mapping was based.

### 3.2. FIRM Mapping

FIRM mapping is an invasive endocardial phase mapping technique that utilizes a custom-made, 64 pole basket contact catheter (FIRMap, Topera, Palo Alto, CA, USA; Constellation, Boston Scientific, Marlborough, MA, USA) covering at least 80–90% of the atria to map AF. AF propagation maps, generated from monophasic action potentials and processed using specialized software, are used to guide intra-procedural elimination of sources demonstrating appropriate spatial and temporal stability [54]. The CONFIRM (Conventional Ablation for Atrial Fibrillation With or Without Focal Impulse and Rotor Modulation) trial evaluated 97 AF patients (72% persistent) undergoing conventional ablation consisting of PVI ± roofline ablation vs. conventional plus FIRM guided ablation [6]. In this study, 97% of patients demonstrated temporally stable rotors or focal impulses with a significantly greater number seen in persistent compared to paroxysmal AF patients. Ablation of these drivers resulted in AF termination or slowing in 86% of participants and at a median follow-up of 273 days, single procedure freedom from AF was significantly greater in patients undergoing adjunct FIRM guided ablation (82.4% vs. 44.9%, *p* < 0.001). These results were maintained at three years [57] leading the authors to conclude that this novel mechanistic-based ablation technique may offer a promising future treatment paradigm for AF. Nevertheless, other groups have failed to replicate the success of the CONFIRM trial with success rates of <40% demonstrated in further studies with this approach [58,59]. Furthermore, early results from the REAFFIRM trial, the only prospective, randomized trial comparing adjunct FIRM guided ablation to conventional PVI failed to demonstrate a benefit in 375 persistent AF patients with similar single procedure success rates at 1 year (69.3% vs. 67.5%, *p* = 0.96) [60].

### 3.3. ECGi Mapping

**Figure 1:**
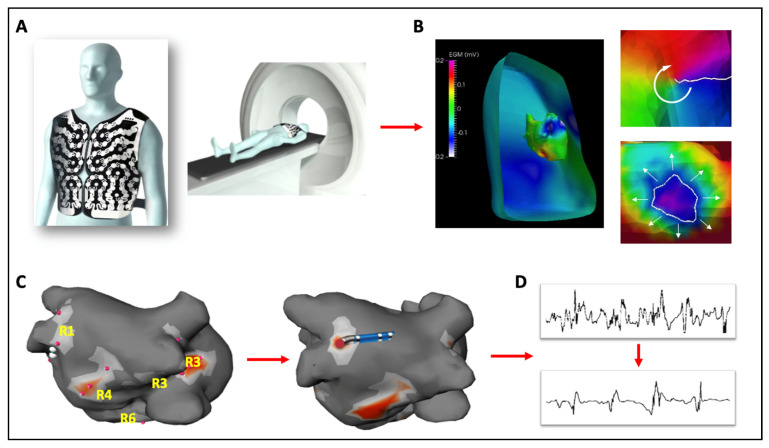
ECGi mapping approach. A 252 body-surface electrode array, combined with thoracic CT imaging (**A**) allows for the depiction of virtual cardiac potentials on the epicardial surface (**B**) and can identify re-entrant (**B**, right top panel) and focal activities (**B**, right bottom panel) from activation maps generated from unipolar electrograms. The AFACART study used this technique to identify driver sites in both atria which were targeted for ablation (**C**) with the endpoint of converting complex, fractionated signals into simple, discrete signals (**D**). R1-R6 indicate regions where CFAEs were identified.

Electrocardiographic imaging mapping or ECGi mapping is a non-invasive phase mapping approach that utilizes a 252 body-surface electrode array combined with thoracic imaging to display virtual cardiac potentials on the epicardial surface (Figure 1). Re-entrant and focal activities are identified from activation maps generated from unipolar electrograms combined with phase mapping analysis. This technique was first applied by Cuculich et al. in 2010 in a study of continuous bi-atrial activation mapping in human AF validated against invasively generated CARTO maps [61]. In this study, multiple wavelets were identified as the most common pattern of activation in 92% and ablation at critical sites on ECGi resulted in restoration of sinus rhythm. Subsequently, using the commercially available ECVUE mapping system (CardioInsight, Cleveland, OH, USA), the Bordeaux group described unstable re-entry circuits with varying spatio-temporal activity (in contrast to the stable focal sources demonstrated on FIRM mapping) as the predominant sustaining mechanism in 103 persistent AF patients [62,63]. In patients with ablation-induced AF termination, arrhythmia-free survival was 87% at one year, similar to a comparison group undergoing the stepwise approach. In 2017, the multicentre AFACART study reported on the efficacy of ECGi guided ablation in persistent AF patients, in eight European centres, using AF termination as a primary endpoint [64] (Figure 1). Ablation strategy consisted of targeted ablation of drivers and PVI, with further linear ablation if AF persisted. In total 4.9 ± 1.0 driver sites were mapped per patient with 53% located in the left atrium and 27% in the right atrium. Driver-only ablation resulted in AF termination in 64% of patients with 77% free from AF at one year. It is worth noting however that no randomized trial exists to date evaluating this technology in AF ablation.

Inherent limitations of these mapping techniques relate to electrode density and mapping resolution as well as the potential for poor electrode contact in the case of invasive FIRM mapping. Transformation of electrograms using phase mapping is a complex process, and an obvious disadvantage is a limited ability for raw signal analysis by the operator prior to transformation. This, coupled with conflicting results regarding driver stability using FIRM vs. ECGi mapping has fueled skepticism about reproducibility and validity. In recent years, further systems have been developed to facilitate mapping of focal and rotational activity without the need for phase mapping transformation including the Cartofinder (Biosense Webster) contact mapping system and the AcQMap (Acutus Medical) non-contact ultrasound-based mapping system. These platforms are capable of identifying focal and rotational activation during AF with the elimination of such activity associated with high procedural AF termination and midterm success rates of up to 72% in persistent AF patients [65,66]. As for ECGi mapping, no randomized trials exist for these techniques.

Taken together, the aforementioned studies do not suggest a significant incremental benefit of an adjunct ablation strategy targeting rotors or drivers, with arrhythmia-free survival similar to a conventional ablation approach. Further development in mapping technology and standardization of electrogram analysis is needed to facilitate ongoing understanding of AF mechanisms that can be translated into a meaningful clinical benefit to the patient.

## 4. Novel Technology for Catheter Ablation

Returning to the central message of STAR AF II, and considering the overall modest results reported with mechanistic mapping techniques, in recent years focus has been placed on the biophysics of ablation and the durability of ablation lesions. Although the inferior results seen in persistent AF ablation may be explained by an incomplete understanding of AF mechanisms, another possible explanation is a failure to achieve durable transmural ablation lesions at index procedure. Certainly, prior to the introduction of contact force sensing catheters, rates of pulmonary vein reconnection at repeat procedure were reported as high as 94% [67] while significantly lower rates were seen with contact force sensing technology [68,69]. Nevertheless, the multicentre, randomized TOUCH-AF trial failed to demonstrate an outcome benefit to contact force guided ablation in persistent AF patients, although gaps in lesion sets were associated with significantly less contact force and a lower force–time integral [70]. Contact force, however, represents only one metric of ablation lesion assessment and the force-time integral does not account for power delivery to the myocardium. As such, in recent years more complex models of lesion prediction have been developed.

### 4.1. Optimized Workflows for Ablation

The Ablation Index (Carto3, Biosense Webster) is a real-time lesion assessment index incorporating force, time, and power in a weighted formula. Ablation Index has been shown in canine studies to predict lesion depth [71] and in humans to identify sites of pulmonary vein reconnection at repeat procedure [72]. Lesion contiguity is also crucial to pulmonary vein reconnection [73]. High single procedure success rates have been demonstrated using a novel workflow, ‘the CLOSE protocol’, focused on the creation of contiguous (interlesion distance ≤ 6 mm), optimized RF lesions with targeted Ablation Index values (550 anterior, 400 posterior) in patients with paroxysmal AF [74,75]. The PRAISE-AF study assessed this protocol in 44 patients with persistent AF undergoing first-time PVI with a protocol-mandated repeat procedure at 2 months [76]. Pulmonary vein reconnection was seen in 22% of patients at repeat procedure and ablation at the intervenous carina required in 44% to achieve durable PVI. At one year 95% of patients were in sinus rhythm leading the authors to conclude that high clinical success rates can be achieved in most persistent AF patients using optimized AI guided PVI alone, re-emphasizing the central role of PVI in current guidelines.

### 4.2. Novel Techniques for Substrate Ablation

#### 4.2.1. Optimized RF Lesions for Linear Ablation

**Figure 2:**
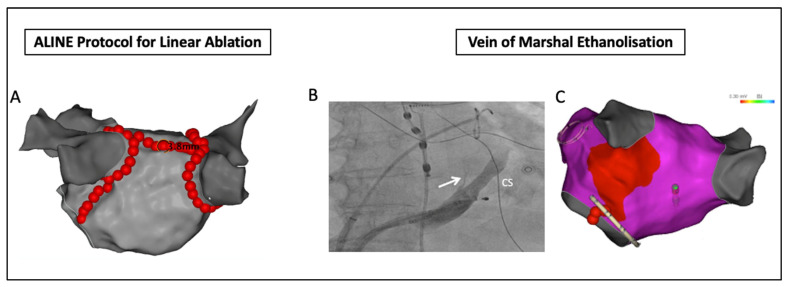
Strategies for substrate ablation in persistent AF. (**A**) Roof and mitral isthmus lines created using optimized RF lesions as per the ALINE protocol. (**B**) Fluoroscopic image of contrast injection of the coronary sinus with the Vein of Marshall indicated by the white arrow. (**C**) Voltage map post ethanolization of the Vein of Marshall demonstrating the resultant area of low voltage and additional endocardial lesions at the mitral annulus performed to achieve mitral isthmus block. CS = coronary sinus.

Aside from PVI, novel RF delivery protocols have been employed for, and shown benefit in, additional substrate ablation in patients with persistent AF. The ALINE study examined the effect of optimized, contiguous RF lesion delivery (interlesion distance ≤ 6 mm, ablation index ≥ 550) on the rate of first-pass block of linear ablation at the left atrial roof and mitral isthmus in 41 patients with persistent AF. A high rate of first-pass block at the roof (93%) but not the mitral line (23%) was reported using this protocol [77] (Figure 2). Additional endocardial and epicardial RF applications resulted in a final rate of bidirectional mitral isthmus block of 80%, underscoring the challenges with RF ablation alone at this site.

#### 4.2.2. Vein of Marshall Ethanolization

Indeed, the complex anatomy of the mitral isthmus often necessitates additional epicardial ablation within the coronary sinus in order to achieve block. Ethanol infusion of the Vein of Marshall was proposed as an adjunct to RF ablation with several studies confirming its added value for achieving block along the mitral isthmus (Figure 2). A 2020 comparative study of 262 patients demonstrated a significantly higher rate of acute mitral isthmus block (98.7% vs. 63.6%) with adjunct Vein of Marshall ethanolization compared to RF only, with more durable block at repeat procedure [78]. A further publication from the same group again emphasized the high rate of mitral isthmus block achievable (98%) with Vein of Marshall ethanolization in combination with RF ablation endocardially and within the great cardiac vein [79]. Aside from its role in facilitating block at the mitral isthmus, the Vein of Marshall has been additionally implicated as a source of AF triggers and the site of parasympathetic and sympathetic nerve fibers important in the pathogenesis and maintenance of AF [80,81]. Emphasising this point, the recently published VENUS-AF trial randomized 343 patients with persistent AF to catheter ablation alone vs. catheter ablation plus Vein of Marshall ethanol infusion and reported significantly greater freedom from AF or atrial tachycardia at 6 and 12 months post-procedure [82]. Secondary analysis identified the presence of peri-mitral block as a significant determinant of post-procedural success [83]. Nevertheless, arrhythmia-free survival was modest (65.2% vs. 53.8%) with a significant amount of additional substrate ablation performed in both groups. While the rate of mitral isthmus block is undoubtedly improved with this technique, further work is needed to fully elucidate any associated incremental benefit on outcomes in the persistent AF population.

#### 4.2.3. Voltage Guided Ablation

Invasive assessment of atrial bipolar voltage has gained significant attention over the last number of years, with left atrial scarring, defined as a bipolar voltage of <0.05 mV in sinus rhythm, identified as a powerful independent predictor of AF recurrence post ablation [23]. In recent years several groups have evaluated strategies of voltage guided ablation through ablation or isolation of low voltage regions with favourable outcomes, albeit in small numbers. Rolf et al. reported similar success rates for those with low voltage areas undergoing voltage guided ablation compared to patients without low voltage areas undergoing standard ablation [84]. In 2016, Cutler et al. demonstrated an improvement in outcome when voltage maps were used to guide additional ablation along the left atrial posterior wall in a small retrospective study [7]. The STABLE-SR multicentre study randomized patients to PVI and homogenization of low voltage areas or the stepwise approach with similar success rates (74% vs. 71%, *p* = 0.325) albeit with lower procedure and fluoroscopy times in those undergoing the voltage guided approach [85]. Endpoints and ablation strategies vary between studies however and large-scale trials with clear and standardized endpoints are needed to ascertain the added value of this approach.

### 4.3. Cryoablation for AF

In the last decade, cryoablation, the ‘single-shot’ technique of isolating the pulmonary veins using a cryoballoon cooled to approximately −40 °C has emerged as an alternative catheter ablation strategy in patients with AF [86]. Its non-inferiority to a conventional RF ablation approach in both safety and efficacy was demonstrated in the Fire and Ice multicentre randomized controlled trial in paroxysmal AF patients [87]. More recently, the multicentre STOP Persistent AF study supported its safety and feasibility in the persistent AF population with 12-month freedom from AF of 54.8% [88]. Further multicentre registry data demonstrated a 61% single procedure freedom from AF at a median of 2.4 years in the persistent AF cohort [89]. The Fire and Ice II randomized trial will compare outcomes between cryo and RF ablation in persistent AF patients and will be important in determining the ongoing role of the technique in this population [90].

### 4.4. Outcomes Measures and Proposed Workflow for Persistent AF Ablation

Given the variable success rates reported in prior studies of ablation outcome, it is important to consider the methods employed for post-procedural rhythm monitoring when interpreting results. The intensity of rhythm monitoring on follow-up has been demonstrated in multiple studies to be associated with greater sensitivity and specificity for the detection of arrhythmia recurrence [91]. This is of relevance given the considerable variability between studies regarding the timeframe, duration, and method of follow-up monitoring. While earlier studies tended to employ 24-h Holter monitoring at 3–6 month intervals for detection of recurrence, in recent years there has been a trend towards more intensive monitoring with 7-day to 1-month monitoring or continuous monitoring with implantable loop recorders.

Although procedural success has been traditionally defined as freedom from ≥30 s of atrial tachyarrhythmia, the CLOSE to CURE study evaluating ‘CLOSE’ protocol guided ablation utilized the novel outcome measure of atrial tachyarrhythmia burden on implantable cardiac monitoring. In this study, a 100% reduction in arrhythmia burden was demonstrated on follow-up [74]. This redefinition of endpoints may be of relevance to the persistent AF population in whom a significant reduction in atrial arrhythmia burden undoubtedly represents a more clinically meaningful outcome than traditionally employed endpoints.

**Figure 3:**
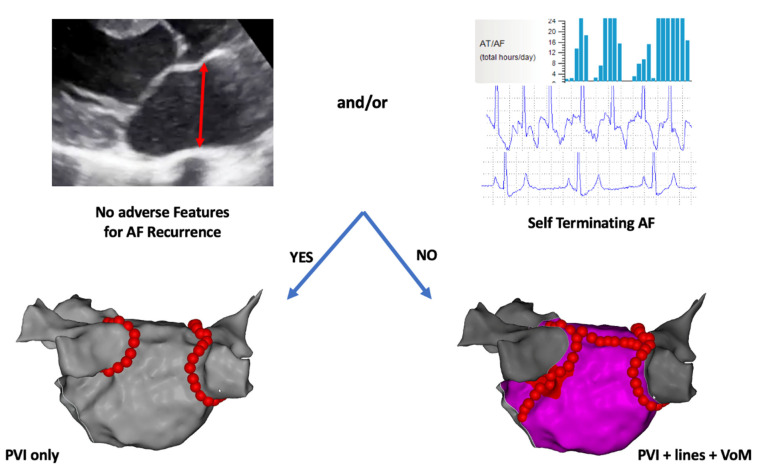
Proposed ablation strategy for persistent AF. Patients with no adverse features for recurrent AF (left atrial dilatation, fibrosis, etc.) and/or evidence of self-terminating AF on continuous monitoring undergo PVI only while those with adverse features or non-self-terminating AF undergo PVI plus linear ablation at the roof and mitral isthmus facilitated by Vein of Marshall ethanolization. VoM = Vein of Marshall (ethanolization).

The broad spectrum of atrial remodeling encompassed by the term persistent AF suggests that ‘patient-tailored’ ablation may be a more appropriate strategy than ‘one size fits all’ ablation in this cohort. In our centre, therefore, we undertake a workflow for first-time persistent AF ablation that is guided by AF burden on continuous long-term monitoring and the presence of risk factors for non-PV triggered AF. Those with self-terminating AF and no features of advanced atrial remodeling are deemed to have ‘pseudo’ persistent AF and undergo PVI alone as per the CLOSE protocol [3]. Patients not fulfilling these criteria are deemed to have truly persistent AF and undergo PVI plus substrate modulation consisting of a roof and mitral isthmus line using the ALINE criteria facilitated by Vein of Marshall ethanolization (Figure 3). This protocol is being evaluated as part of the ongoing CLOSEMAZE study with atrial tachyarrhythmia burden on continuous monitoring as a primary endpoint. Preliminary data suggest that one-third of recruited patients with persistent arrhythmia, resistant to anti-arrhythmic medication and requiring at least one cardioversion, demonstrate self-terminating AF. This again underscores the diversity of the AF substrate amongst patients with persistent AF and the need for a better understanding of AF mechanisms and optimization of substrate assessment in order to guide treatment on a more individualized basis.

## 5. Future Perspectives

### 5.1. High Power–Short Duration Ablation

High power–short duration ablation is a novel technique that offers a theoretical advantage due to a reduction in the time-dependent conductive heating phase of lesion formation with less consequent collateral tissue damage [92]. In swine, high power–short duration ablation at 90 Watts results in more contiguous lesions than conventional ablation with a wider diameter but shallower depth [93]. In humans, the single centre Power-AF study demonstrated greater procedural efficiency and similar midterm efficacy in paroxysmal AF patients undergoing CLOSE protocol guided PVI at 45 W vs. 35 W [94]. The QDOT Fast multicentre study evaluating very high power ablation demonstrated the feasibility of 90 W/4 s ablation, again in paroxysmal AF patients [95]. A significant oesophageal complication occurred in both studies; however, suggesting a narrower margin of safety with increased power at the posterior wall. Ongoing studies will shed light on safety concerns and the potential role for this novel ablation technology in expanded populations including those with persistent AF.

### 5.2. Pulsed Field Ablation

One of the most exciting developments in ablation delivery in recent years is that of pulsed field ablation (PFA), a non-thermal ablative technique that causes cell death through the destabilization of cell membranes. Its potential advantage over RF lies in the preferential targeting of myocardial tissue with a reduction in collateral tissue damage demonstrated in pre-clinical studies [96,97,98]. The first-in-human IMPULSE and PEFCAT trials evaluating the Farapulse PFA system and multi-spline catheter (Farapulse, Menlo Park, CA, USA) in 81 patients with paroxysmal AF reported 100% PVI durability at three months along with an excellent safety profile [99]. Subsequently, the same group performed an evaluation of this technology in patients with persistent AF using the multi-spline PFA catheter for PVI and posterior left atrial wall ablation [100]. Isolation of the pulmonary veins and the posterior wall was achieved in all patients and re-evaluated at 75 days post-procedure. In all cases, the posterior wall remained isolated, as did 96% of pulmonary veins. The favorable safety and efficacy profile demonstrated in this small study led the authors to conclude that PFA may have a promising role in the treatment of persistent AF patients. Ongoing trials in this field include the PULSED AF and INSPIRE trials; prospective, non-randomized, multicentre studies evaluating the safety and efficacy of the Medtronic PulseSelect platform for PFA delivery and the Biosense Webster PFA system and VARIPULSE catheter respectively. Initial results from the PULSED AF trial report 100% acute pulmonary vein isolation with no significant safety concerns [101]. Further technological development has allowed for combined PFA and RF delivery using a novel expandable lattice catheter which has shown promise for short-term efficacy and safety in patients undergoing PVI and linear ablation at the LA roof, mitral isthmus, and CTI [102,103]. Although large-scale trials are needed for further assessment, this combined approach may have application in persistent AF patients in whom additional substrate modification is required and may not be achieved with a PFA catheter alone. Data on safety and mid-term outcomes from large-scale trials will be essential to fully ascertain the role of this exciting technology going forward and the patient groups likely to derive the greatest benefit from it.

## 6. Conclusions

Patients with persistent AF remain a particularly challenging cohort with conflicting evidence regarding the optimal catheter ablation strategy in these patients. Progressive atrial remodeling beyond the pulmonary veins results in substrate-driven AF that is likely to be mechanistically different from that seen in paroxysmal disease, with further significant variation in the degree of remodeling within the persistent AF cohort itself. Despite the wealth of published data on adjunctive substrate-based ablation for persistent AF, no technique has demonstrated a consistent benefit over PVI alone, and latest guidelines underscore the central role of PVI in all patient populations. Advances in catheter technology coupled with optimized, parameter-driven ablation workflows have translated into increased PVI durability on follow-up in recent years, with novel methods for energy delivery demonstrating further potential for improving ablation efficacy and safety. This progress coupled with ongoing research into AF mechanisms and advanced substrate assessment will be central to the refinement of ablation strategy on a more individualized basis and the improvement of outcomes in this population.

## Data Availability

Not applicable.
